# Breath-hold CT attenuation correction for quantitative cardiac SPECT

**DOI:** 10.1186/2191-219X-2-33

**Published:** 2012-06-22

**Authors:** Kazuhiro Koshino, Kazuhito Fukushima, Masaji Fukumoto, Kazunari Sasaki, Tetsuaki Moriguchi, Yuki Hori, Tsutomu Zeniya, Yoshihiro Nishimura, Keisuke Kiso, Hidehiro Iida

**Affiliations:** 1 Department of Investigative Radiology, National Cerebral and Cardiovascular Center Research Institute, 5-7-1 Fujishirodai, Suita, Osaka 565-8565, Japan; 2 Department of Radiology and Nuclear Medicine, National Cerebral and Cardiovascular Center Hospital, 5-7-1 Fujishirodai, Suita, Osaka, 565-8565, Japan; 3 Graduate School of Medicine/Faculty of Medicine, Osaka University, 1-1 Yamadaoka, Suita, Osaka, 565-0871, Japan

## Abstract

**Background:**

Attenuation correction of a single photon emission computed tomography (SPECT) image is possible using computed tomography (CT)-based attenuation maps with hybrid SPECT/CT. CT attenuation maps acquired during breath holding can be misaligned with SPECT, generating artifacts in the reconstructed images. The purpose of this study was to investigate the effects of respiratory phase during breath-hold CT acquisition on attenuation correction of cardiac SPECT imaging.

**Methods:**

A series of ^201^Tl-emission and ^99m^Tc-based transmission computed tomography (TCT) scans was carried out along with CT-attenuation scans on 11 young normal volunteers using a hybrid SPECT/CT scanner. The CT scans were performed at three respiratory phases: end-inspiration (INS), end-expiration (EXP), and the midpoint (MID) between these phases. Using alignment parameters between attenuation maps and SPECT images without attenuation or scatter corrections, quantitative SPECT images were reconstructed, including corrections for attenuation and scatter. Regional radioactivity concentrations normalized by the subjects’ weights were compared between CT- and TCT-based attenuation correction techniques.

**Results:**

SPECT images with CT attenuation maps at the EXP phase showed significant differences in regional weight-normalized radioactivity concentrations relative to the images using the other attenuation maps (*p* < 0.05), as well as systematic positive bias errors, compared to TCT-based images for all myocardial segments, 5.7% ± 2.7% (1.9% to 10.0%). No significant differences in regional weight-normalized radioactivity concentrations were observed between images with CT attenuation maps at MID and INS phases or between these and the TCT-based images, but regional tendencies were found: for anterior to anterolateral segment, positive bias of 5.0% ± 2.2% (1.3% to 8.1%) and 5.6% ± 1.9% (2.6% to 8.5%) and for inferior to inferoseptal segment, negative bias of −5.3% ± 2.6% (−9.1% to −1.7%) and −4.6% ± 2.5% (−8.8% to −1.5%) for the MID and INS phases, respectively.

**Conclusions:**

Use of breath-hold CT attenuation maps at INS and MID phases for attenuation and scatter corrections demonstrated accurate quantitative images that would prove beneficial in cardiac SPECT/CT studies.

## Background

Single photon emission computed tomography (SPECT) has the unique ability to provide functional images of biological tissues *in vivo*. As is the case with positron emission tomography (PET), corrections for attenuation and scatter are important. Previous studies have demonstrated improvements in diagnostic accuracy when applying corrections for both attenuation and scatter [1]. Attenuation correction has been widely carried out by means of transmission computed tomography (TCT) scanning, with several mechanical configurations such as rotating rod sources fitted with parallel-beam collimator systems, and a single rod source placed at the focal line of a symmetrical fan-beam collimator [2-4]. It has been demonstrated that TCT data can also be used for scatter correction [5], and that when coupled with kinetic analysis, the overall accuracy of reconstructed SPECT images is sufficiently high for quantitation of various physiological functions in the thoracic region [6-8] and also in the brain [9]. Several dedicated TCT systems developed by various manufacturers have resulted in improved diagnostic accuracy. It has also been reported that when using optimally designed TCT equipment, reconstructed images are accurate and applicable to quantitative studies in the thoracic region [10].

Myocardial blood flow (MBF) quantitation has been shown to be feasible using ^201^Tl and dynamic SPECT if proper procedures are applied to accurately correct for attenuation and scatter [8]. It has been demonstrated that a kinetic modeling approach can be applied to quantitatively obtained SPECT data to determine regional myocardial blood flow. In fact, in a canine study with correction for all necessary factors, MBF values assessed at rest, after beta-blocker administration, and during adenosine infusion were in good agreement with those determined in a microsphere experiment [11]. Based on these previous works, it is likely that correction for attenuation and scatter is an important key for the establishment of absolute MBF measurement in cardiac SPECT.

Recently, an X-ray computed tomography (CT)-based method of attenuation correction has been implemented with commercial PET cameras and now has become standard as a combined PET/CT system. This type of system has been used primarily not only in oncology studies but also for the quantitation of myocardial perfusion using ^13^ N-ammonia [12]. Combination of multi-slice CT and SPECT systems has been shown to result in accurate reconstruction in myocardial studies [13]. A problem, however, has been noted when applying CT information to attenuation correction in the thoracic region. Erroneous observations occur and are attributed to misregistration between CT and PET images. These are associated not only with global patient movement but also with respiratory motion. Previously, ^68^Ge-based TCT scans were carried out for 3 to 10 min at each bed position and, thus, provided spatially and temporally smoothed images that were consistent with the emission data, resulting in no systematic artifacts in reconstructed PET images. On the other hand, the CT-based method, which provides frozen images during one respiratory phase, can cause significant mismatches with PET data and, thus, cause artifacts, often in the apex and lateral wall regions [14].

The same problem has also been noted with combined SPECT/CT systems [15,16]. These studies recommended careful review of attenuation correction maps as well as software-based re-registration in order to avoid reconstruction artifacts due to misregistration.

In cardiac studies with PET/CT, it would be ideal to match cine CT to PET images at each phase [17] or to use respiratory-averaged CT information as the attenuation map for reconstructing single-frame PET data sets [18]. However, these techniques require large radiation doses for multiple CT scans, resulting in their limited use in clinical studies. In contrast, it has been noted that the use of CT images acquired at specific phases provides reasonable accuracy in producing attenuation-corrected PET images [18].

The purpose of this study was to investigate the effect of respiratory phase on breath-hold CT-based attenuation correction (CT-AC) for quantitative cardiac SPECT imaging. The CT-based attenuation maps were acquired from normal volunteers at three respiratory phases: end-inspiration (INS), end-expiration (EXP), and the midpoint (MID) between these phases. The attenuation maps were used for quantitative SPECT (QSPECT, National Cerebral and Cardiovascular Center and QSPECT group, Japan), namely for scatter correction using transmission-dependent convolution subtraction technique followed by ordered-subset expectation maximization (OSEM) reconstruction with attenuation correction [8,19]. The CT-corrected emission images were compared with images reconstructed using ^99m^Tc TCT-based attenuation maps, which have been validated in a previous article [10].

## Methods

### Subjects

Subjects consisted of 11 healthy volunteers (7 men, 4 women; age range, 18 to 25 years; mean age ± SD, 21.4 ± 2.3 years; mean weight ± SD, 55.4 ± 5.2 kg). The volunteers had no signs or symptoms of ischemic heart disease. The study protocol was approved by the ethics committee of the National Cerebral and Cardiovascular Center. Prior to the scan, all subjects gave written informed consent for participation in this study.

### Image acquisition

**Figure 1:**
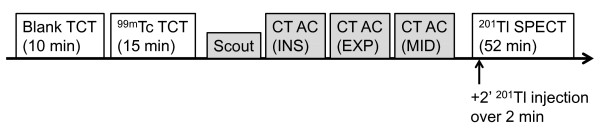
**Protocol for cardiac SPECT/CT and**^**99m**^**Tc TCT.** INS, EXP, and MID denote respiratory phases at end-inspiration, end-expiration, and the midpoint between these phases, respectively.

All subjects were placed in supine position with arms up and scanned at rest using a hybrid SPECT/CT scanner, the Symbia T6 (Siemens, Knoxville, TN, USA). As shown in Figure 1, the study protocol consisted of ^99m^Tc-based blank and TCT scans, X-ray CT scans, and a dynamic SPECT.

**Figure 2:**
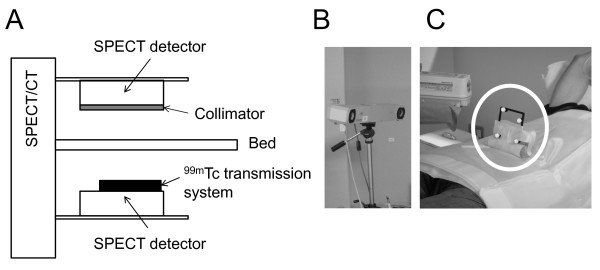
**Experimental setup.** (**A**) Schematic diagram of ^99m^Tc transmission system attached to a SPECT/CT detector. (**B**) and (**C**) System to monitor respiratory motion; (B) optical motion-tracking device. (C) Infrared-reflective target placed on the subject’s abdominal surface.

A 10-min blank scan and a 15-min TCT scan, used as references for CT-AC, were performed using a TCT system validated in a previous work [10]. A small scatter fraction with maximum sensitivity was achieved by optimizing the collimator gap for the ^99m^Tc source. The system was attached to an opposing two-headed detector with low-energy, high-resolution (LEHR) collimators, as shown in Figure 2A. The TCT source consisted of a 3-m long tube (5 mm in diameter) filled with 740 MBq of ^99m^Tc, which was equivalent to seven separate rod sources. Each rod was collimated axially with lead sheets. The TCT system was removed from the SPECT detector before the following scans.

After acquisition of a CT scout image, breath-hold CT-AC scans were performed at the INS, EXP, and MID respiratory phases. CT acquisition parameters were as follows: helical scan time, 10 s; rotation cycle, 1 s; pitch, 1.0; tube voltage, 130 kVp; tube current, 100 mA over 10 s; slice thickness, 5 mm; slice interval, 5 mm; matrix size, 512 × 512; pixel size, 0.98 × 0.98 mm^2^; and maximum duration for holding breath, 16 s. For breath-hold CT acquisition at each phase, a system of monitoring and displaying respiratory phase was introduced. Briefly, a subject’s abdominal respiratory motion was determined using an optical motion-tracking device, POLARIS (Northern Digital Inc., Ontario, Canada), to monitor the location of an infrared-reflective target placed on the abdominal surface, as shown in Figure 2B,C. The accuracy of the device was 0.35-mm root mean square (RMS), as reported by the manufacturer. The motion was displayed on a screen using an ellipsoidal indicator to enable the subject to control his or her own breathing in real time. The subject breathed deeply several times to determine the marker’s range of motion between the EXP and INS phases before the CT-AC scans. The MID phase was defined as occurring when the marker was located at the central position of the motion range.

Dynamic SPECT begun 2 min before the start of the 2-min constant infusion of 111-MBq ^201^Tl. Projection data were acquired using the detector heads positioned opposing each other (H-mode) with the LEHR collimators in continuous mode and in a circular orbit because QSPECT reconstruction was performed on geometric-mean projections. The frame collection rates and 360° rotation times were 6 × 2 min (28 s) and 8 × 5 min (148 s); number of views, degrees per view, matrix size, and enlargement factor were 45 views, 4° per view, 64 × 64, and 1.45, respectively. A 34% energy window centered on 77 keV was used for the ^201^Tl acquisitions [7,20].

### Image reconstruction

SPECT projection data were processed to generate a quantitative image, that is, pixel values were calibrated in Bq/mL. The procedure was as follows: (1) TCT projections normalized by blank projections and CT projections were reconstructed and then linearly scaled to provide attenuation maps for ^201^Tl. For CT images, filtering to SPECT spatial resolution using the B08s filter, which was called by the manufacturer, interpolation to equate the matrix and pixel sizes to those of TCT attenuation map, and conversion from Hounsfield units to attenuation coefficients were performed using Syngo MI Application, version 7.0.7.14 (Siemens, Knoxville, TN, USA). (2) A SPECT image was temporarily reconstructed using a filtered backprojection algorithm without scatter or attenuation correction. Each attenuation map was manually aligned to the SPECT image using translations along three orthogonal directions by interactively moving the attenuation map image over the SPECT image. The interactive fusion was performed using the QSPECT software package. The overlap in the aligned images was assessed in coronal, sagittal, and transaxial views based on the distances of myocardial contours between the anterior to lateral regions of the attenuation map and the SPECT images. (3) Using each alignment parameter, scatter- and attenuation-corrected images were reconstructed with OSEM (three iterations, five subsets using geometric-mean projections, post-reconstruction Gaussian filter of 7.0 mm in full width at half maximum) provided by the QSPECT software package [8], respectively.

### Data analysis

Each dynamic image reconstructed using the four attenuation maps was summed into a static image with the 14.5- to 34.5-min frames (mid-scan time). Transformation to a short-axis orientation was based on a static image corrected with a TCT attenuation map (TCT-AC image). The transformation was also applied to the static images corrected with CT attenuation maps (CT-AC images). The radioactivity concentrations of the images were normalized by the subject’s weight. Variability of normalized radioactivity concentrations in the myocardium was also assessed. Polar maps for the left ventricles were generated from the static images. In this process, the basal slice and apical region were defined on the TCT-AC image. The definition was also used for the CT-AC images of the same subject. In addition, percent differences between the weight-normalized radioactivity concentrations of TCT-AC and CT-AC images were calculated as $100\times \frac{\text{CT}-\text{TCT}}{\text{TCT}}$. Regional radioactivity concentration values and percent differences were evaluated for myocardial segments by dividing polar maps into 17 segments according to the AHA 17-segment model [21]. To evaluate the effects of attenuation correction with the four attenuation maps on SPECT images, the regional radioactivity of TCT-AC and CT-AC images was statistically tested using the Tukey multiple comparisons method. Homogeneity of radioactivity distribution among the 17 myocardial segments was also assessed using the Tukey multiple comparisons method for each dataset of TCT-AC and CT-AC images. A *p*-value of less than 0.05 was considered statistically significant.

## Results

**Figure 3:**
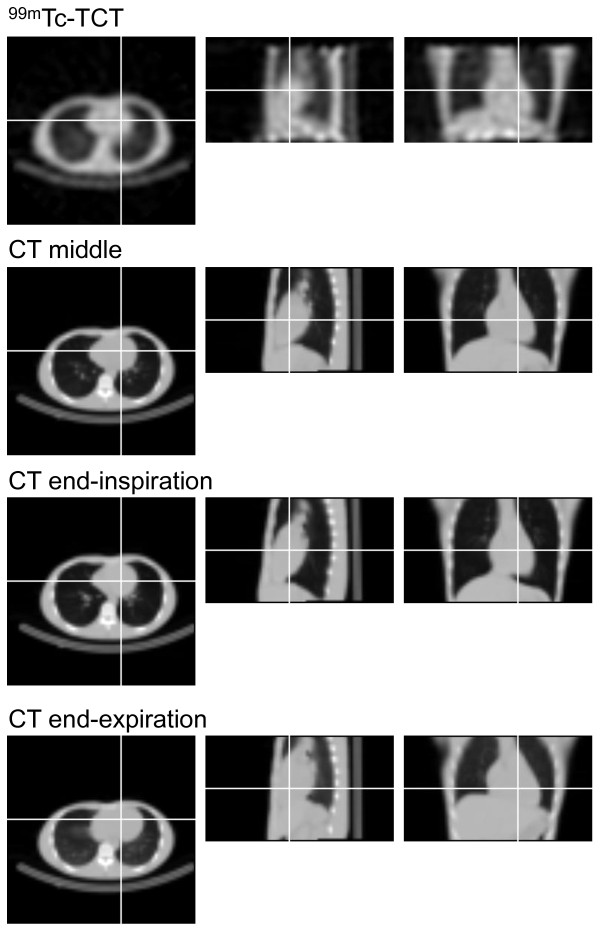
**Attenuation map images of**^**99m**^**Tc-TCT and CT at three different respiratory phases.** The left, middle, and right columns represent transaxial, sagittal, and coronal views, respectively.

**Figure 4:**
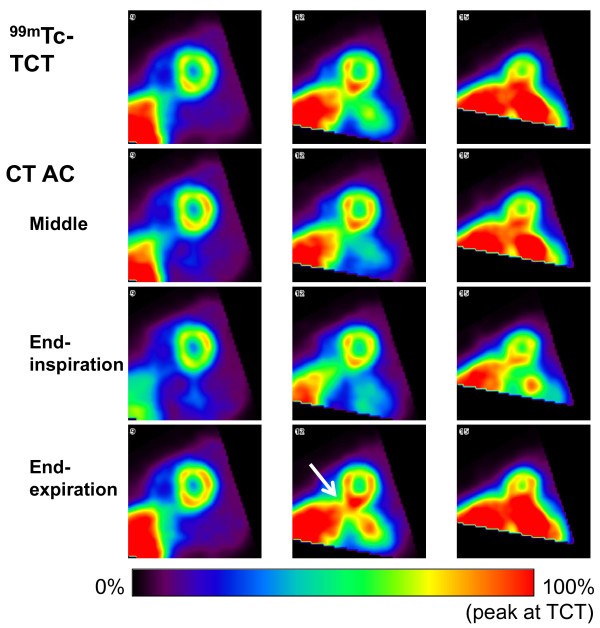
**SPECT images corrected for attenuation and scatter using TCT and CT attenuation maps.** The left, middle, and right columns represent slices at the basal, middle, and apical levels, respectively. The subject was the same as in Figure 3.

Breath-hold CT-AC scans at three respiratory phases were performed successfully for all subjects. The amplitude of the marker indicating respiratory phases, which was defined as half the distance between the infrared-reflective targets at the INS and EXP phases, was 8.50 ± 5.58 (2.51 to 17.50) mm. Translations to align attenuation maps to SPECT image was listed in Table 1. Cephalad/caudal translations were dominant among three orthogonal directions, especially in CT attenuation maps at the MID and the EXP phases. Figure 3 shows examples of TCT and CT attenuation map images at the three phases. In coronal and sagittal views at the INS phase, the surfaces of the inferior regions of the myocardium and the liver were clearly separated. On the other hand, at the EXP phase, it was difficult to distinguish these surfaces. The position of the heart relative to the liver at the MID phase seemed to be similar to that of TCT attenuation map rather than those at the other respiratory phases. Figure 4 shows SPECT images for the same subject as Figure 3. The left, middle, and right columns represent slices at basal, middle, and apical levels, respectively.

**Table 1:** Translations to align attenuation maps to SPECT image (mean ± SD)

**Attenuation map**	**Direction**		
	**Right/left (mm)**	**Up/down (mm)**	**Cephalad/caudal (mm)**
TCT	0.0 ± 0.0	0.0 ± 0.0	2.4 ± 2.1
CT at MID	2.8 ± 7.1	−2.6 ± 3.9	11.7 ± 8.0
CT at INS	4.5 ± 6.0	−4.7 ± 5.4	6.2 ± 7.3
CT at EXP	−1.5 ± 3.5	0.0 ± 5.1	19.9 ± 5.8

CT, computed tomography; EXP, end-expiration; INS, end-inspiration; MID, midpoint; TCT, transmission computed tomography.

**Figure 5:**
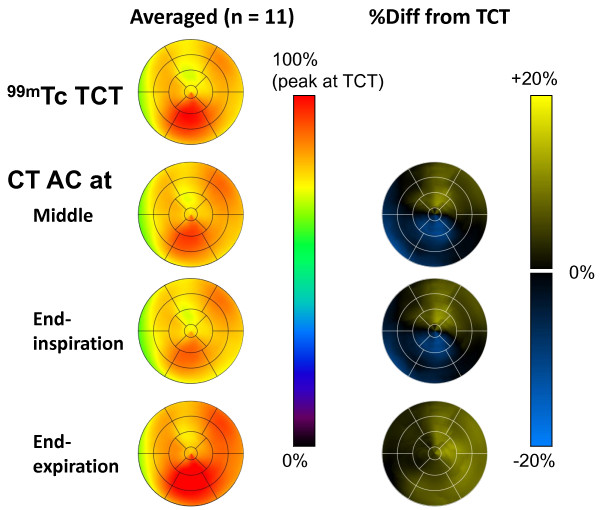
Polar maps of averaged radioactivity concentrations and percent differences from images corrected with TCT attenuation maps.

The distribution of weight-normalized radioactivity concentration and the differences between TCT-AC and CT-AC images are shown in Figure 5. The left column displays polar maps of averaged radioactivity concentration, normalized using the maximum value of the TCT-AC map. Statistical analysis of the SPECT image datasets with the four attenuation maps indicated that the images corrected with EXP attenuation maps were different significantly from the other datasets, that is, significant differences in weight-normalized radioactivity concentrations were observed between TCT-EXP, INS-EXP, and MID-EXP image datasets (there was no significant difference in weight-normalized radioactivity concentration between TCT-INS, TCT-MID, and INS-MID). No regional differences were found in weight-normalized radioactivity concentrations of any datasets, although relatively large values in inferior regions were observed for all TCT-AC and CT-AC image datasets. As shown in the right column, the polar map of the percent difference for CT-AC images of EXP phases shows a positive bias, 5.7% ± 2.7% (1.9% to 10.0%), over all segments. Regional tendencies were found for CT-AC images of the other two phases: for the anterior to anterolateral segments, positive biases of 5.0% ± 2.2% (1.3% to 8.1%) and 5.6% ± 1.9% (2.6% to 8.5%) and for the inferior to inferoseptal segments, negative biases of −5.3% ± 2.6% (−9.1% to −1.7%) and −4.6% ± 2.5% (−8.8% to −1.5%) for the MID and INS phases, respectively. The worst segmental percent differences (mean ± SD and the worst individual value) were 10.5% ± 4.9%, 22.6% in the basal inferolateral segment for the EXP phase; −4.8% ± 4.8%, −11.0% and −8.0% ± 6.4%, −18.4% in the mid inferior segments for the MID and the INS phases, respectively. The percent difference excluding the worst individual value was 9.3% ± 3.0% in the basal inferolateral segment for the EXP phase. It was considered that the relatively large differences in radioactivity concentrations of the EXP polar map, as shown in Figure 5, compared to the MID or the INS polar maps were due to the results over all subjects, but was not from outliers.

## Discussion

This study investigated the effects of respiratory phases on breath-hold CT-based attenuation correction in cardiac SPECT with monitoring of the respiratory phase and amplitude. Reconstruction using CT attenuation maps at the INS and MID phases, with correction for attenuation and scatter, provided quantitative SPECT images that agreed with images derived from TCT-based attenuation maps. The SPECT images with CT attenuation maps at INS and MID phases showed similar radioactivity concentrations, as seen in Figures 4 and 5. This may be due to similarities between CT attenuation maps at INS and MID, for instance the separation between the surfaces of the inferior myocardial wall and the liver, as shown in Figure 3. Polar map analysis of the four attenuation maps showed no significant heterogeneity in any image dataset in terms of weight-normalized radioactivity concentrations.

The CT-AC images corrected with EXP attenuation maps differed significantly from the TCT-AC and CT-AC image datasets at INS and MID phases. In addition, for all segments, the percent differences from TCT-AC images indicated a bias of CT-AC images with EXP attenuation maps: a positive bias in anterior to anterolateral segments and a negative bias in inferior to inferoseptal segments for CT-AC images with both INS and MID attenuation maps, as shown in Figure 5. The magnitude of the bias for CT-AC images with EXP attenuation maps was larger than that of CT-AC images with the other respiratory attenuation maps. As previously reported, MBF was non-linearly related to the radioactivity concentration for ^201^Tl kinetics [8,11]. Error with positive bias in the concentration propagated to error in MBF values more than negative bias with the same magnitude. Therefore, we considered the CT attenuation maps at the INS or MID phases to be preferable to those at the EXP phase. Instead of breath-hold CT acquisitions, respiratory gating in both SPECT and CT scans was expected to be another approach for CT-AC. Uniformity of radioactivity concentrations in myocardia has been improved by respiratory-gated SPECT acquisition [22]. Although respiration-related artifacts must be corrected ideally, the breath-hold CT-AC might be suitable for dynamic SPECT studies with low-dose injections, such as measurement of absolute MBF, which needed temporal changes in radioactivity concentrations.

Although no significant heterogeneity in regional radioactivity concentrations was observed, Figures 4 and 5 show reduced radioactivity concentrations in the anterior regions near the apexes and increased concentrations in the inferior regions in images with all attenuation maps. Potential reasons for the reduced concentrations are as follows: first, residual errors in registration between an attenuation map and a SPECT image. Fricke et al. reported that a 3.5-mm misalignment in the ventrodorsal direction induced a spurious defect in the anteroseptal wall in a phantom study [23]. The second reason is spillover from the anterior wall due to partial volume effects. The anterior wall was surrounded by void space in terms of ^201^Tl concentration. Spillover from the wall, particularly near the apex, could be affected by the partial volume effects three-dimensionally. In contrast, possible reasons for relatively high concentrations in the inferolateral to inferior wall are as follows: (1) residual errors in registration as seen with the anterior wall, (2) overestimation of attenuation effects around the inferolateral to lateral walls caused by the liver, as pointed out by Cook et al. [18], and (3) spillover from other organs. Little effect of overlap between the heart and the liver was reported by McQuaid and Hutton [24]. However, in their numerical phantom study, spatial resolution effects of a SPECT scanner were excluded, and the assumed activity ratio between the myocardium and the liver was 75:30. Their experimental conditions were different from those in our human study, specifically the spatial resolution of the SPECT scanner and the activity ratio of the two organs (liver/anterolateral myocardium = 1.1 ± 0.3, range = 0.7 to 1.7). High radioactivity concentration was also observed in the left kidney (the activity ratio of the left kidney/anterolateral myocardium = 1.5 ± 0.3, range = 1.1 to 2.0). It could cause additional spillover to the anterolateral myocardium. Anatomical information from the CT image prior to spatial resolution matching to SPECT was expected to help correct for the spillover and, thus, produce a more accurate quantitative SPECT image.

In this study, the use of CT-AC attenuation maps with breath holding was validated at resting conditions. In order to measure coronary flow reserve, a stress SPECT scan is needed. A series of resting and stress scans could take a relatively long time. For optimal comfort, the patient would step off the scanner bed during the interval between the two scans, or the two scans would be carried out on different days. In such cases, additional CT-AC scans would be needed for accurate attenuation correction. The respiration amplitude observed by the monitoring system was 8.50 ± 5.58 (2.51 to 17.50) mm. The accuracy of the system, 0.35-mm RMS, was considered sufficient to discriminate the three respiratory phases during a single session, that is, while a subject lay continuously on the scanner bed. However, it would be difficult, using the monitoring system, to ensure inter-subject reproducibility of the amplitude of respiratory motion as well as intra-subject results obtained during different sessions. The respiratory motion was estimated by measuring positions of the infrared-reflective target placed on the abdominal surface. The amplitudes and directions of surface movements depend on the individual subject, the location of the target, and any non-linear deformation of the abdominal surface and internal organs.

The registration of an attenuation map and a pre-reconstructed SPECT image, prior to reconstruction of a quantitative SPECT image corrected for attenuation and scatter, was limited to translations in three spatial directions. If a rigid-body model including rotations or a non-linear registration was employed, an image corrected with CT-AC at the EXP phase might provide a consistent result with a TCT-based scan or CT-AC at the other respiratory phases. However, the use of INS or MID attenuation maps has an advantage over the EXP attenuation map in terms of separation between the heart and the liver surfaces, which would contribute to accurate registration of attenuation maps and pre-reconstructed SPECT images. As shown in Table 1, translations toward caudal directions were needed to align CT attenuation maps at any respiration phases to SPECT images. The reason could be different baseline positions of the hearts during breath-hold CT acquisitions and SPECT acquisitions with breathing freely, as previously reported by Pan et al. [25]. For TCT attenuation map, no alignment between the attenuation map and SPECT image was needed essentially because temporal resolutions of TCT and SPECT scans for respiratory motions were nearly identical. The slight shifts of TCT attenuation maps to SPECT images in cephalad/caudal directions, 2.4 ± 2.1 mm, might be caused by global and/or intra-torso motions of subjects.

## Conclusion

Use of breath-hold CT attenuation maps at end-inspiration and middle phases for attenuation and scatter corrections demonstrated accurate quantitative images in cardiac SPECT/CT studies. This technique might be applicable to routine clinical study. Quantitative assessment of absolute MBF and coronary flow reserve in clinical settings would be an additional potential application.

## Competing interests

The authors declare that they have no competing interests.

## Authors’ contributions

KaK participated in the development of a respiratory motion indicator system and analysis of SPECT data. KS participated in the image processing for reconstruction of SPECT images. YH participated in the measurement of respiratory motions. TM and TZ participated in the discussion of spillover effects and misregistration effects between emission and attenuation data and helped to draft the manuscript. MF and YN carried out SPECT/CT studies as radiological technologists. KF and KeK participated as physicians. HI conceived the application of the breath-hold CT to the cardiac SPECT study and helped to draft the manuscript. All authors read and approved the final manuscript.
